# Correction of a Class III Malocclusion with a Functional Shift and Severe Crowding

**DOI:** 10.1155/2020/8867130

**Published:** 2020-11-24

**Authors:** Yahya A. Alogaibi, Ahmad A. Al-Fraidi, Manar K. Alhajrasi, Ali A. Hassan

**Affiliations:** ^1^Bisha Dental Center, Ministry of Health, P.O. Box 418, Bisha 61922, Saudi Arabia; ^2^Department of Orthodontic, King Fahad Hospital, Specialized Dental Center, Madina, Saudi Arabia; ^3^Department of Orthodontic, North Jeddah Specialty Dental Center, MOH, Jeddah, Saudi Arabia; ^4^Alfarabi Private College, Jeddah, Western Region, Saudi Arabia; ^5^Department of Orthodontics, Faculty of Dentistry, King Abdulaziz University, P.O. Box 80209, Jeddah 21589, Saudi Arabia

## Abstract

A forward functional shift of the mandible is a significant problem that can cause both functional and aesthetic complications for many patients. This shift usually occurs in growing patients, and it is unusual to see in adult patients. This case report shows an adult patient with a forwarding functional shift that caused both anterior and posterior crossbites with a pseudo class III dental and skeletal relationship. The patient also showed severe upper arch crowding with blocked-out canines and mild crowding in the lower arch. The treatment of this patient involved extraction of the upper right and left first premolars and the lower right first premolar, followed by opening of the bite to relieve the neuromuscular reflex of the forward protrusion of the mandible during centric occlusion and to correct both the anterior and posterior crossbites. Extraction spaces were closed using class III elastics and elastomeric chains. At the end of the treatment, good functional and aesthetic results were obtained after the elimination of the forward functional shift.

## 1. Introduction

Class III malocclusions can be classified into three main types: pseudo class III, dental class III, and skeletal class III malocclusions. The first to suggest such classification was Tweed who, in 1966, categorized class III malocclusions into two categories: skeletal or pseudo class III [1]. The pseudo class III malocclusion was described as forward displacement of the mandible during closure accompanied by an anterior crossbite [2].

Skeletal class III malocclusions, on the other hand, have many characteristic features that may include some or all of these features: deficiency in the midface, upward and forward rotation of the mandible, prominence of the lower lip, and protruded mandible [3, 4]. The main difference between pseudo and skeletal class III malocclusions is the ability of the pseudo type to retrude the mandible in the class I relation, showing a normal appearance of the mandible without any obvious protrusion. Also, it can be diagnosed by observing the forward functional shift of the mandible during closure from the point of initial contact with the teeth, up to complete centric occlusion [5]. This is why it is sometimes called a forward functional shift as it only occurs during function (i.e., biting in occlusion) and not during rest. In most cases, these patients exhibit anterior crossbites in two or more incisors [6].

Treatment of these cases requires the correction of the neuromuscular reflex that causes this shift. This is usually done by either anterior or posterior bite-opening devices, which allow the mandible to assume its normal functional position without any forward shift [7, 8].

However, it is unusual to find this forward functional shift in adult patients as this problem usually progresses to a skeletal problem due to the effects of muscles on the growth of the mandible [2, 9, 10].

This case report describes an unusual case of an adult patient with a pseudo class III malocclusion that manifested as a forward functional shift during centric occlusion combined with retroclined upper incisors and severe upper anterior crowding.

## 2. Diagnosis and Treatment Planning

A 21-year-old female patient was referred to the orthodontic department with a chief complaint of inability to bite properly on her teeth. No significant problems were found in her medical history. Her dental history, on the other hand, showed a history of extraction of the lower left 1^st^ molar and multiple restorations. Extraoral examination revealed a mesocephalic facial type, straight facial profile, and competent lips with a fairly symmetrical and well-proportioned face. Intraoral findings showed severe crowding in the upper arch that caused both upper canines to be blocked out, an anterior crossbite that included all of the upper incisors with a -3 mm reverse overjet, and a 1 mm shift to the left side in the upper midline. A posterior crossbite with cusp-to-cusp occlusion was also seen with a molar (class III) on the right side and the absence of the lower 1^st^ molar on the left side. The lower arch showed a very mild degree of crowding and mesioangular impaction of the lower right third molar. However, when the patient assumed the rest position of the mandible, an edge-to-edge bite was obtained with a class I molar relation, which strongly suggests a forward functional shift of the mandible. Smile analysis showed an asymmetrical soft tissue smile with a flattened smile arc. No gingival tissues were exposed during smiling (low lip line), and no canting of the occlusal plane or buccal corridor was shown upon smiling. The thin periodontium was also noted (also called the washboard effect), which increases the risk of gingival recession (Figures 1 and 2).

Cephalometric analysis revealed a class III skeletal base and normal inclination of the mandibular plane with normal vertical proportions. However, when another cephalometric analysis was made in the rest position of the mandible (i.e., centric relation), the skeletal relation was shown to be a normal class 1 relation. Regarding dentoalveolar measurement, it was found that the upper and lower incisors had normal inclinations (Figure 3, Table 1).

Panoramic radiography showed a missing lower first molar on the left side, multiple restorations, endodontic treatment of many teeth, and mesioangular impaction of the lower right third molar (Figure 3).

Additionally, in the area of the lost first molar, a radiopaque shadow was noted that may signify the presence of chronic focal sclerosing osteomyelitis (also called condensing osteitis) which was related to the molar as a bone reaction to a previous periapical infection in this area. Another differential diagnosis for this radiopaque shadow includes idiopathic osteosclerosis, and both of these lesions do not require any treatment.

### 2.1. Treatment Objectives

The following treatment objectives were planned: (1) reinforce adequate oral hygiene and professional fluoride application before commencing treatment; (2) correct the anterior crossbite via protrusion of the upper incisors, retraction of the lower incisors, and backward shift of the mandibular arch to be enclosed inside the maxillary arch and correct the posterior crossbite via expanding the archwire; (3) achieve class I molars and canines on both sides except on the left molars, in which a class II molar relation will be achieved; (4) correct the upper midline shift and alleviate crowding; and (5) retain the achieved results.

### 2.2. Treatment Plan

Two treatment options were available:

The first option is to extract the upper right and left first premolars and the lower right first premolar. Then, open the bite with Build-Up composite resin on the molars followed by correction of the crossbites and space closure. The type of anchorage will be minimal in both the upper and lower arches.

The second option is to extract the upper right and left canines and the lower right first premolar and to substitute the upper canines with the upper first premolars. Then, continue with the treatment as in the first option.

After discussion of the current findings with the patient and considering the priorities of the aesthetic and functional demands for this patient, the first option was approved.

### 2.3. Retention Plan

The retention protocol was fixed retainers from 3-3 to wraparound retainers in the upper and lower arches in order to avoid any relapse, allow for teeth settling, maintain the expanded arch, and help in closing the band spaces.

### 2.4. Treatment Progress

The treatment was initiated by banding the first molars and the lower left second molar and bonding of the other teeth using 0.018 slot preadjusted edgewise ceramic brackets with Roth prescriptions. A letter for referral to extract the upper first premolars and the lower right first premolar was given to the patient, and an atraumatic extraction of these teeth was done. Build-Up composite resin was applied on both of the lower molars. Leveling and alignment were done by using a 0.012^″^ NiTi wire, followed by 0.014^″^ NiTi, 0.016^″^ NiTi, 0.016 × 0.022^″^ NiTi, and finally 0.016 × 0.022^″^ stainless steel wires for space closure. Space closure was done by using class III elastics and elastomeric chains. Finishing and detailing were done by using 0.016 × 0.022^″^ and 0.017 × 0.025^″^ stainless steel wires and intermaxillary elastics. All the third molars were extracted except the lower left one. The total duration of treatment was 23 months (Figure 4).

### 2.5. Treatment Results

Remarkable enhancement in the masticatory functions, combined with better facial aesthetics, of the patient was established. Proper and stable intercuspation between the upper and lower teeth was achieved. Both the anterior and posterior crossbites were eliminated. The forward functional shift of the mandible disappeared. However, the upper incisors were more proclined, whereas the lower incisors were more retroclined. Skeletal and dental class I relation was achieved except on the left molar side, where a firm class II molar relation was reached. Periodontal problems and several root resorptions were found; in particular, the upper incisors and buccal corridor were altered. Most importantly, the psychological and physical health of the patient was greatly enhanced (Figures 5–9) (Table 1).

## 3. Discussion

Functional shifts of the mandible can occur in any direction. These may be lateral functional shifts, which cause unilateral posterior crossbites, or forward functional shifts, which cause pseudo class III malocclusions [11, 12]. Forward functional shifts are usually associated with both anterior and posterior crossbites. This usually occurs for two reasons. The first reason is that the occlusal interference causes some of the upper anterior teeth (due to their lingual eruption) to interact with the lower teeth, hindering the normal closure of the mandible in the normal centric relation. The second reason, which is the main cause for the posterior crossbite, is the wider posterior width of the mandibular arch, compared to the anterior width; i.e., when the mandible is advanced forward, its wider posterior arch occludes with the narrower anterior part of the maxilla, causing a posterior crossbite [6, 13–15].

The forward functional shift of the mandible was corrected by bite opening. This was done to remove the neuromuscular response that the mandible was conditioned to during the presence of the occlusal interferences. This was in agreement with a study conducted by Adly et al. who showed that all types of functional shifts totally disappeared after bite opening and the mandible assumed its normal functional position automatically, even before the removal of the occlusal interferences [5, 16, 17].

Extraction of the upper first premolars was done to relieve the severe crowding in the upper arch and allow the canine to assume its normal position. The extraction of the upper canines was avoided because of the ability of the upper canines, with their long roots, to support the bone under the nasolabial sulcus and thus to avoid deepening of this sulcus, which may contribute to an aged appearance of the face. Also, the canine is a strong tooth that is able to achieve a firm and stable canine-guided functional occlusion [18–21].

It is well known that orthodontic treatment can greatly enhance a patient's psychological and physical health [22, 23]. This is due to the better perceived self-image and the increased efficiency of mastication and digestion of food [24, 25]. This was clearly seen in this patient who had remarkable changes in her mental and physical attitudes by being more social and eating healthy foods.

## 4. Conclusion

In this case report, it was found that the forward functional shift of the mandible can be efficiently managed by proper diagnosis and treatment planning; however, the most important step in the treatment of such a case is to diagnose the dental and skeletal relation without this forward shift and build the treatment plan upon this condition as this condition is mostly temporary and resolved by removal of the interferences. The common error in treating these cases is the assumption of the stability of this forward functional shift and building a treatment plan upon this assumption. This may lead to dramatic results with total failure to achieve a stable occlusion.

## Figures and Tables

**Figure 1:**
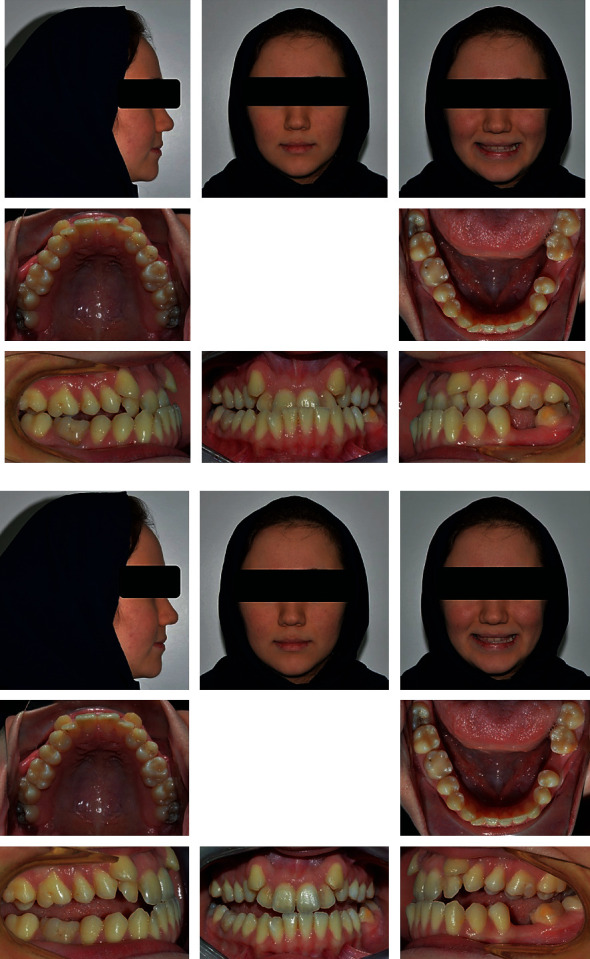
Pretreatment extraoral and intraoral photographs of the patient.

**Figure 2:**
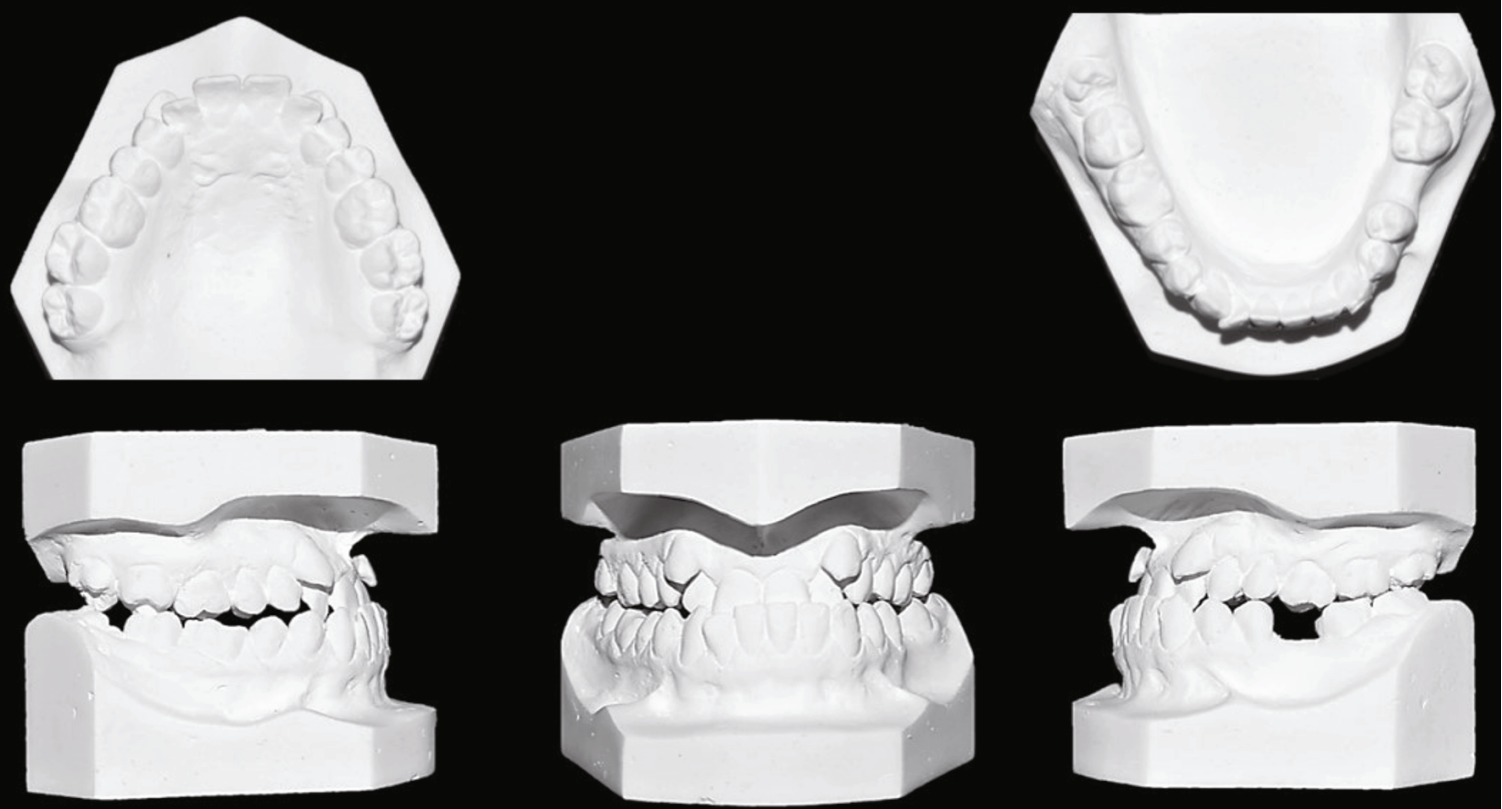
Pretreatment models.

**Figure 3:**
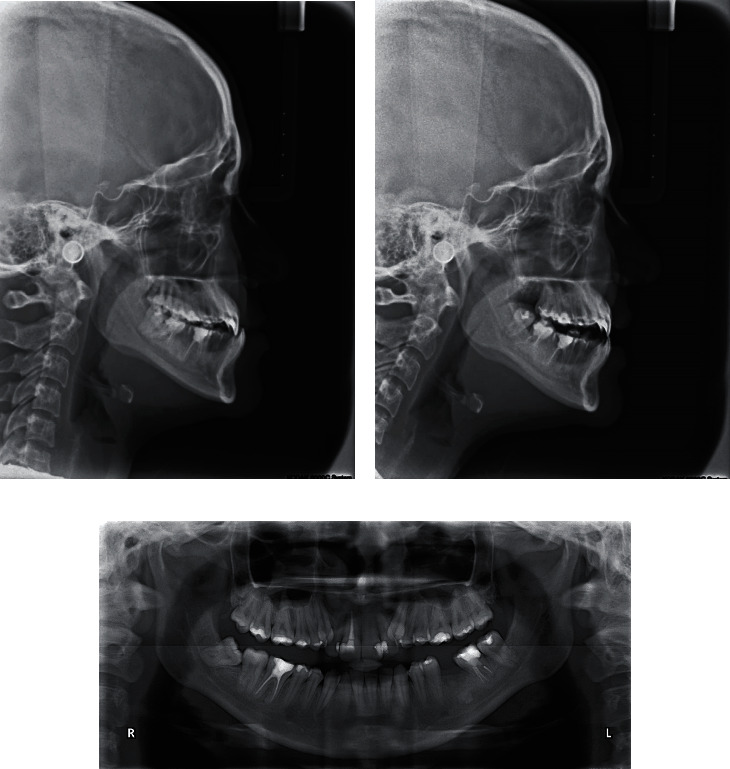
Pretreatment cephalometric and panoramic radiographs.

**Figure 4:**
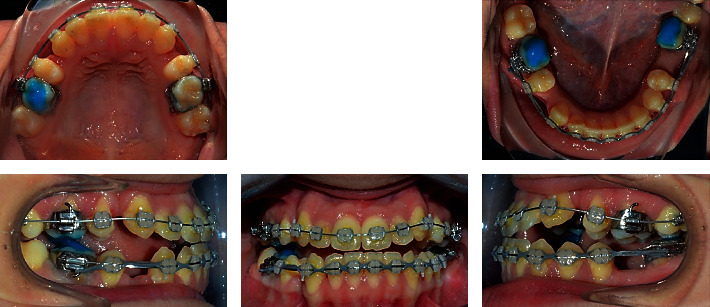
Progress photographs.

**Figure 5:**
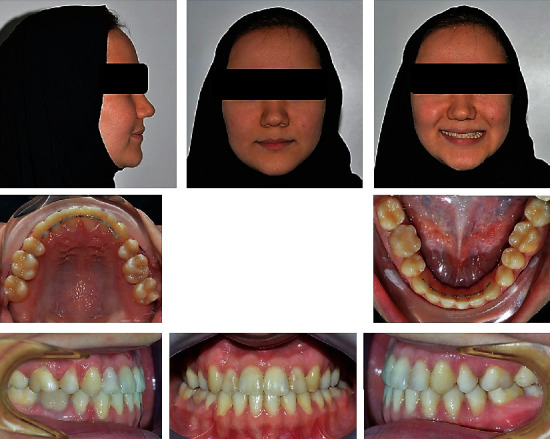
Posttreatment photographs of the patient.

**Figure 6:**
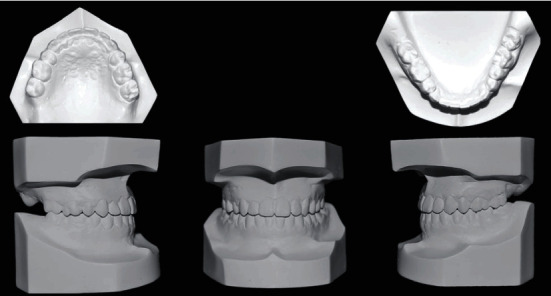
Posttreatment models.

**Figure 7:**
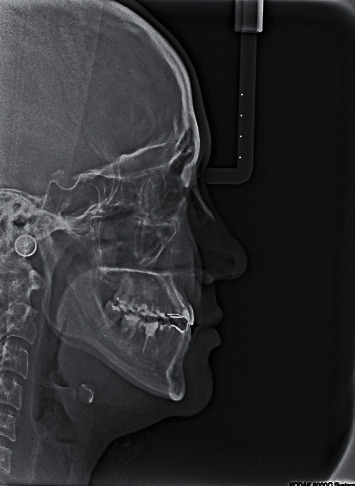
Posttreatment cephalometric radiograph.

**Figure 8:**
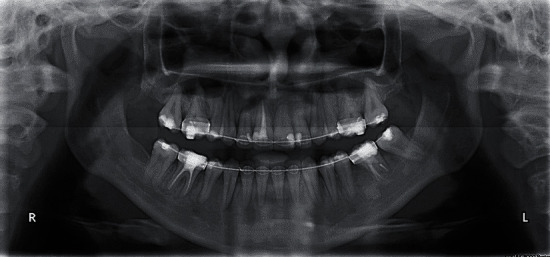
Panoramic radiograph.

**Figure 9:**
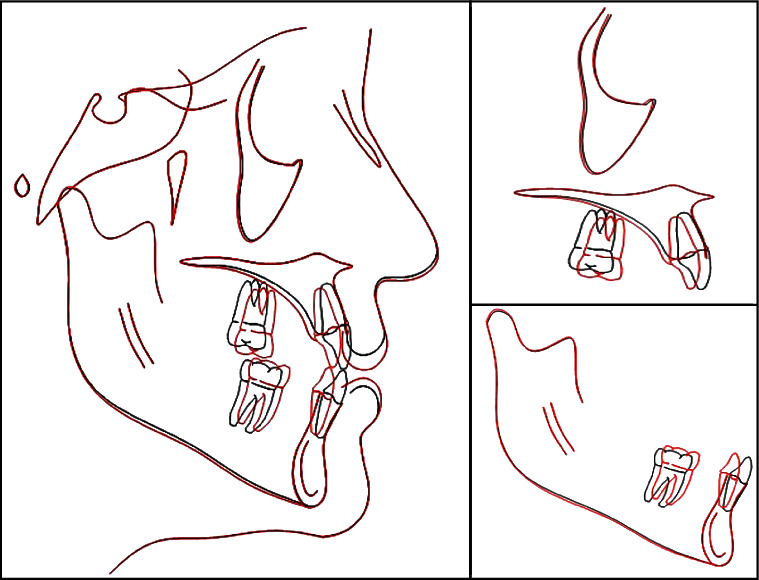
Posttreatment cephalometric superimposition. Black line: pretreatment. Red line: posttreatment.

**Table 1:** Pre- and posttreatment cephalometric measurements.

	Measurement	Mean (±SD)	Saudi norms	Patient	
				Initial	Final
Anteroposterior	SNA (°)	82° (±3.3)	80.8 (±4.06)	77.6	78.8
	SNB (°)	80° (±3.1)	77.5 (±4.48)	79.4	77.4
	ANB (°)	2° (±1.7)	3.7 (±1.5)	-1.8	1.4
	Wits (mm)	*M* = −1.17 (±1.9) *F* = −0.10 (±1.77)	0.13 (±2.47)	-4.9	-1 mm
	Angle of convexity NA-APg (°)	0° (±5.1)	5.01 (±3.05)	11	8
	A-B plane AB:NPg (°)	-4.6° (±3.7)	-4.6° (±3.7)	4	1

Vertical	MP (Go-Gn):SN (°)	32° (±3.5)	35.9 (±5.96)	44	41
	MP (tangent lower border):FH (°)	21.9° (±3.2)	28.5 (±4.79)	41	36
	Pg:NB (mm)	4 (±2)	4 (±2)	0.1	0.1
	*Y*-axis (SGn:FH)	59.4° (±3.8)	69.6 (±4.2)	71	70
	LAFH (ANS to Gn ÷ N to Gn)	0.57 (±0.02)	0.57 (±0.02)	0.57	0.6
	OP:SN (°)	14° (±4.1)	14° (±4.1)	11.2	12
	OP:FH (°)	9.3° (±3.8)	9.3° (±3.8)	8.1	8.7

Dental	U1 to palatal plane (°)	109° (±6)	109° (±6)	108	112
	U1 to NA (°)	22° (±6.1)	27.3 (±7.5)	16.8	27.5
	U1 to NA (mm)	4 (±1.2)	6.8 (±2.9)	2	3
	L1 to NB (°)	25° (±4.5)	29.34 (±6.98)	31	35.5
	L1 to NB (mm)	4 (±1.5)	7.52 (±2.63)	3	4
	U1 to L1 (°) (Avg. Downs & Steiner)	131.7° (±6.5)	120 (±11.98)	124	113
	U1:APg (mm)	2.7 (±1.8)		1.3	2.5
	L1:APg (mm)	1 (±2)		3.1	3.9
	FMA (°)	25° (16-35)		50	50
	FMIA (°)	65° (60-75)		41	36
	IMPA (°)	90° (85-95)		89	94

Soft tissue	Facial convexity–G Sn Pg′ (°)	12° (±4)		9	9
	Facial angle (FH:N′Pg′) (°)	90-92°		92	91
	Nasolabial angle (°)	90-110°	80.8 (±4.06)	103	103
	Esthetic plane (E-line)—upper lip	-4 mm	77.5 (±4.48)	-2	-3
	Esthetic plane (E-line)—lower lip	-2 mm	3.7 (±1.5)	-1	0
