# Corrigendum to “Correction of a Class III Malocclusion with a Functional Shift and Severe Crowding”

**DOI:** 10.1155/2021/9761307

**Published:** 2021-09-01

**Authors:** Yahya A. Alogaibi, Ahmad A. Al-Fraidi, Manar K. Alhajrasi, Ali H. Hassan

**Affiliations:** ^1^Bisha Dental Center, Ministry of Health, P.O. Box 418, Bisha 61922, Saudi Arabia; ^2^Department of Orthodontic, King Fahad Hospital, Specialized Dental Center, Madina, Saudi Arabia; ^3^Department of Orthodontic, North Jeddah Specialty Dental Center, MOH, Jeddah, Saudi Arabia; ^4^Alfarabi Private College, Jeddah, Western Region, Saudi Arabia; ^5^Department of Orthodontics, Faculty of Dentistry, King Abdulaziz University, P.O. Box 80209, Jeddah 21589, Saudi Arabia

In the article titled “Correction of a Class III Malocclusion with a Functional Shift and Severe Crowding” [1], the name of the last author was given incorrectly as Ali A. Hassan. The name should be corrected to Ali H. Hassan, and the revised author list is shown above.

Additionally, in Section 2.2 of the article body, “The type of anchorage will be minimal in both the upper and lower arches” should be corrected to “The type of anchorage will be maximum in the upper arch and minimum in the lower arch.”

There were errors in Figures 4 and 5 due to the images originally being collected using a mirror. For clarity, the revised figures have been flipped to ensure the accurate description of the location of each tooth. The correct figures are as follows.

## Figures and Tables

**Figure 1:**
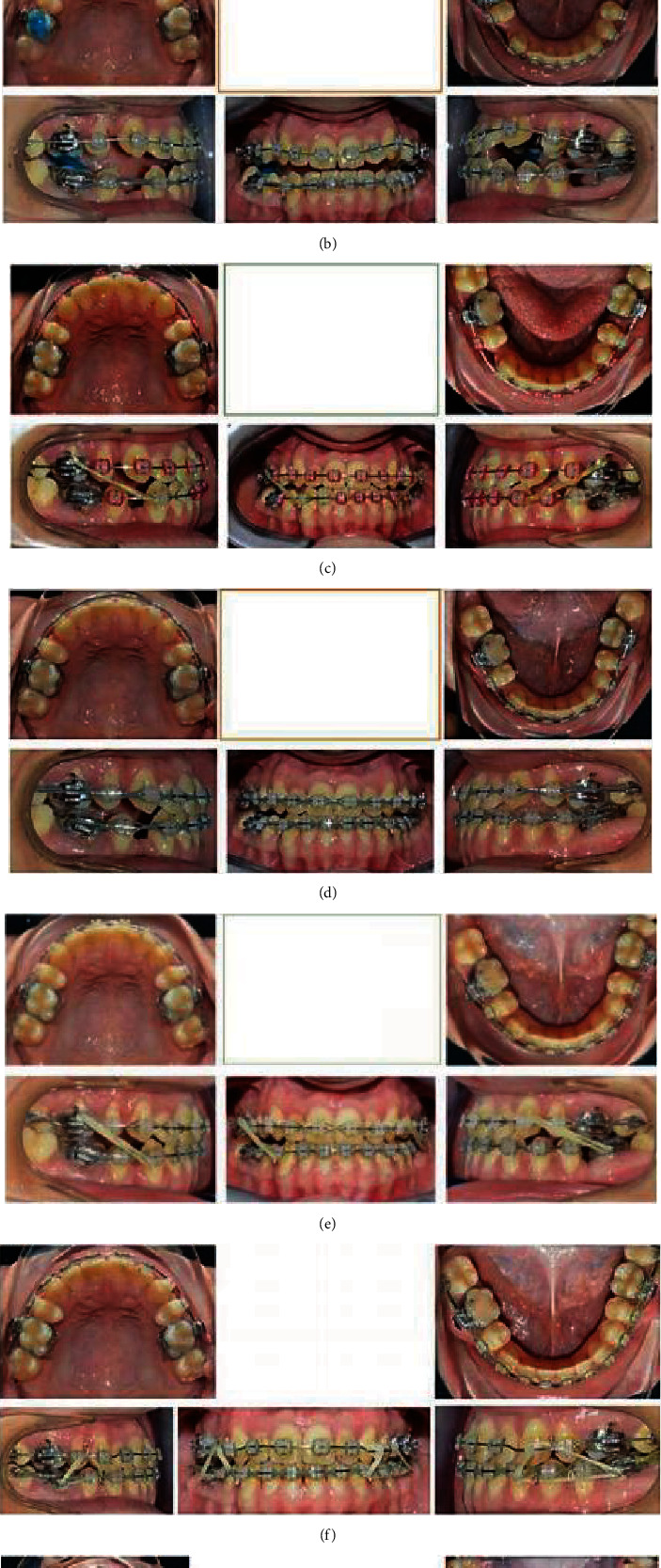
Progress photographs.

**Figure 2:**
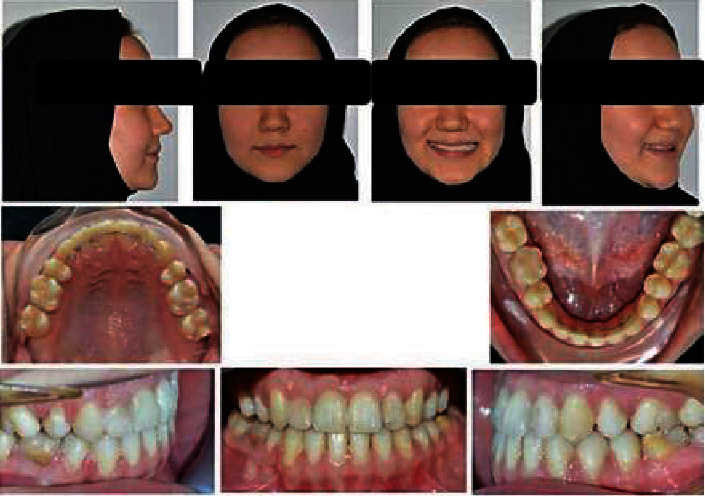
Posttreatment photographs of the patient.
